# Experiential Avoidance Mediates the Association between Emotion Regulation Abilities and Loneliness

**DOI:** 10.1371/journal.pone.0168536

**Published:** 2016-12-28

**Authors:** Rui Shi, Shilei Zhang, Qianwen Zhang, Shaoping Fu, Zhenhong Wang

**Affiliations:** 1 College of Humanities, Northwest A&F University, Yangling, P.R. China; 2 Psychological Counseling Centre, Chang’an University, Xi’an, P.R. China; 3 School of Psychology, Shaanxi Normal University, Xi’an, P.R. China; Wuhan University, CHINA

## Abstract

Experiential avoidance (EA) involves the unwillingness to remain in contact with aversive experiences such as painful feelings, thoughts, and emotions. EA is often associated with the development and maintenance of emotional problems. Since loneliness is characterized by negative emotions such as sadness and pessimism, which is often linked to emotional problems, this study aims to test the mediating effects of EA on the relationship between emotion regulation abilities (ERA) and loneliness in a sample of Chinese adults. Five hundred undergraduates completed questionnaires measuring EA (Acceptance and Action Questionnaire; AAQ-Ⅱ), ERA (Failure-relate action orientation; Action Control Scale, ACS-90) and loneliness (UCLA Loneliness Scale). Structural equation modeling showed that EA fully-mediated the relationship between ERA and loneliness. The findings suggest EA is a key mechanism in explaining why people with high ERA are prone to feeling lower levels of loneliness. In particular, these findings have important implications for designing effective psychological interventions for loneliness.

## Background

Loneliness is an aversive state which reflects a deficit in social connection [1]. According to the evolutionary theory of loneliness, loneliness is momentary in general [2]. However, many longitudinal studies prove that, for some individuals, loneliness can be a prolonged experience with harmful effects on mental and physical health over several years [3–6]. For instance, the chronic feeling of loneliness has been linked to anxiety [7], depression [8] and it is related to individuals’ higher risk for many physical problems, such as cardiovascular disease [9], sleep problems [10], and even suicide [11]. A study by Holt-Lunstad [12] et al. found that loneliness was a risk factor for morbidity and mortality, and the likelihood of death increased 26% for reported loneliness. Since loneliness is an important topic in the sociological, psychological, medical, and nursing literature, an enhanced understanding of loneliness and related phenomena would potentially be beneficial to individuals’ health and well-being.

According to the literature on loneliness, different types of factors can predict loneliness, such as socio-demographic [13], health-related [14], and psychological factors [6]. Recent empirical studies have uncovered a close relationship between emotional skills, such as emotional intelligence, and loneliness [15–17]. The findings showed that poor emotional skills increase loneliness over time [15, 16, 18]. Similarly, if people could effectively perceive and manage negative feelings associated with loneliness, they could successfully overcome loneliness [19]. Thus emotion regulation abilities (ERA) may be an important factor related to loneliness.

### Failure-Related Action Orientation as an Emotion Regulation Ability Difference

According to action control theory, emotion regulation processes can be classified with respect to low versus high ERA (state orientation versus action orientation) at a functional level [20]. During failure-related situations (e.g., dangers or painful experiences), high ERA individuals could be more effective in down-regulating stress and negative emotions compared to low ERA individuals; as a consequence, low ERA persons tend to ruminate about failure-related situations for prolonged periods of time [21] and they tend to be at higher risk of developing psychopathologies such as depression or anxiety disorders [22]. Rumination is defined as thoughts and behaviors that focus a person’s attention on negative moods. Rumination has consistent independent effects on many health risk indicators and outcomes such as loneliness [23]. Thus, ERA might be associated with loneliness. Other literature has indicated that low ERA individuals tend to view themselves primarily as entities detached from the social world [24, 25]. However, how the relationship between ERA and loneliness has not previously been investigated.

It is reasonable to infer that mediators exist between ERA and loneliness. According to the functional perspective of emotion regulation, the ability to disengage from negative emotions in threatening situations is adaptive, because such behavior is consistent with an individual’s own goals including maximizing long-term survival or welfare (20). Previous findings suggest that maladaptive emotion regulation strategy, such as inflexible suppression or avoidance of emotion, could mediate the relationship between ERA and mental health [26]. ERA is also positively related to functional coping strategies (reappraising) and negatively related to dysfunctional strategies (suppression of emotional expression) [26, 27].

Therefore in the current study we are interested in the relationship between ERA and loneliness. Specifically, we want to analyze this relationship by adding a mediator: experiential avoidance (EA), which is an inflexible avoidant emotion regulation strategy.

### Experiential Avoidance as a Mediator

EA is receiving increasing attention in the empirical literature of clinical psychology [28–31]. EA is thought to be critical to the development and maintenance of psychopathology, such as addiction, anxiety, depression, and impulse control disorders [32–37]. It involves the unwillingness to remain in contact with aversive experiences such as painful feelings, thoughts and emotions [31, 32]. Although it seems that attempting to hide or inhibit unpleasant thoughts, feelings, and bodily sensations is the most effective way to control feelings in the short-term, applying those techniques rigidly and inflexibly actually increases the frequency and distress of similar experiences, perhaps with an added sense that one is being disconnected from oneself [38, 39]. Whereas acceptance, “a disposition to accept thoughts and feelings” [40], is the opposite of avoidance and could therefore negatively predict loneliness. Specifically, acceptance predicts over a quarter of the variance in loneliness [41]. Thus, low acceptance (high experiential avoidance) may potentially leave people impaired in social interaction [42]. Lonely people will pay less attention to people in interactions [43] and seem less involved in conversations [44]. Moreover, according to theoretical model of metacognitive therapy, EA is a counterproductive emotion regulation strategy which may lead to social anxiety [45]. Social anxiety is the combination of fear and worry that people experience when they anticipate that they have failed to make a positive impression on others [46]. Since social anxiety and loneliness are correlated states, and social withdrawal and isolation often occur as the result of acute social anxiety [47], we infer EA might be related to loneliness.

Additionally, theoretical literature and empirical research provide support for the potential presence of emotion regulation difficulties in the form of EA [48, 49]. Furthermore, EA relates to other less adaptive emotion regulation strategies such as avoidant coping, thought suppression, stress intolerance, and anxiety sensitivity [50]. But the important distinction regarding EA and these strategies is that EA refers to avoidance strategies being applied rigidly and inflexibly, even when such strategies cause harm in the long term. Additionally, EA could mediate the effects of emotion regulation strategies on mood and distress [51]. A previous study found that EA was negatively correlated with ERA [26]. As EA was associated with both ERA and loneliness, we expect that EA could account for the relationship between ERA and loneliness. Aside from the possible theoretical relationship mentioned above, previous research suggested ERA could be viewed as a trait that is linked to an individual’s ability to regulate emotions and cope with stress [52, 53]. Also, EA can be considered as another coping style largely related to ways of emotion-focused and avoidant coping [50]. Therefore, we assume that EA is a strategy and might be a mediator between ERA and loneliness.

Thus the purpose of this study is, first, to examine if EA is related to loneliness, using a sample of Chinese university students. The second purpose is to investigate if EA mediates the anticipated relationship between ERA and loneliness. Here it is hypothesized that: 1) EA will be positively related to loneliness, 2) ERA will be negatively associated with EA and loneliness, and 3) the relationship between ERA and loneliness will be mediated by EA.

## Method

### Participants and Procedure

Five-hundred-twenty-three undergraduates enrolled in a psychology class were invited to participate in this study. All invitees were from one university in northwestern China, and they were asked to complete a study on individuals’ attitudes toward personality and life. Five hundred participants (366 males and 134 females; mean age = 20.6 years; SD = 0.86) completed the questionnaire to be included in this study’s convenience sample, for a response rate of 95.6%.

Data were collected in March, 2015, using questionnaires. Prior to data collection, ethical approval was provided by a research ethics committee. All participants signed individual consent forms and the questionnaire was administered anonymously. The questionnaire included items on ERA, EA, loneliness and some demographic variables (e.g., gender, age).

### Measures

#### Emotion regulation abilities

We used a Chinese edition of the Action Control Scale (ACS-90) to assess individual differences in ERA [21, 54]. The failure-related subscale consists of 12 failure-related actions which assess an individual’s ability to disengage themselves from undesirable events or unobtainable goals (i.e., people with high ERA), as well as an individual’s inability to deal with undesirable experiences and failure (i.e., people with low ERA). Each item has two response alternatives, for example, "When something really gets me down (a) I have trouble doing anything at all" (people with low ERA were coded as 0); (b) I find it easy to distract myself by doing other things" (people with high ERA were coded as 1). The ACS-90 has sufficient reliability and adequate construct validity across cultures [21, 55], and the reliability coefficient (Cronbach’s alpha) for the failure-related subscale in this study was α = 0.74.

#### Experiential avoidance

The seven-item Chinese version of the Acceptance and Action Questionnaire (AAQ-II; Bond et al. 2011; Cao et al. 2013) was used to assess EA. Participants indicated the extent to which they agreed with each of the seven statements from the AAQ-II, which featured seven-point Likert-type response scales with anchors ranging from "never true" to "always true". All answers were summed to form the EA measure, with lower scores reflecting greater psychological flexibility and therefore lower EA. The AAQ-II has demonstrated reasonable internal reliability in different cultural contexts [40, 56], as well as in the present study (α = 0.85, Cronbach’s alpha).

#### Loneliness

Loneliness was assessed using the Chinese translation of the UCLA Loneliness Scale (Version 3) [57, 58], which consisted of twenty statements measuring loneliness on a 4-point Likert-type response scale ranging from "never" to "always". Responses were summed to provide a total loneliness score. Higher scores reflected higher levels of loneliness. The UCLA Loneliness Scale has previously demonstrated good reliability and validity [59]. In this study, the reliability coefficient of the UCLA Loneliness Scale was α = 0.83 (Cronbach’s alpha).

### Data Analysis

We used SPSS version 18.0 to calculate descriptive statistics and correlations between the variables (there was no missing data). Then we used AMOS version 17.0 to analyze the mediation effects, which used maximum likelihood estimation by two-step procedure [60]. Five indices were employed to assess the goodness of fit of the model [61–65]: the Chi-square statistic (χ^2^), χ^2^/*df* of 2 or less, the Comparative Fit Index (CFI) of .95 or more, the Root Mean Square Error of Approximation (RMSEA) of 0.06 or less, and the Standardized Root Mean Square Residual (SRMR) of 0.08 or less (the path coefficients were accepted as significant at the 0.05 level). Since there were 20 items included in the UCLA Loneliness Scale, in order to control for inflated measurement errors due to multiple items for the latent variable, four item parcels (average scores of the items) were created only for the loneliness constructs (α = 0.843, Cronbach’s alpha). The random algorithm method was adopted for the parceling procedure [66].The procedure of creating the item parcels was conducted as follows: first we decided on four parcels to be created, and then we randomly assigned the items to the parcels. The value of each parcel was used as an indicator, or observed variable, which was the average scores of the five items selected for each parcel.

## Results

### Descriptive Statistics and Correlations

Independent samples t-tests indicated that neither gender nor age significantly affected ERA, EA, or loneliness (*p* > 0.05). As shown in Table 1, all variables were significantly correlated in the predicted directions. ERA was negatively correlated with EA and loneliness, and EA was positively correlated with loneliness.

**Table 1:** Correlations, means, and standard deviations for study variables.

Variable	1	2	3
1.ERA	—		
2.EA	-0.499***	—	
3.L	-0.292 ***	0.560***	—
M	6.43	21.27	42.49
SD	2.84	6.96	8.56

Note. n = 500; *ERA* = emotion regulation abilities; *EA* = experiential avoidance; *L* = loneliness

***p <0.001

### Mediation Analyses

In order to test whether EA might mediate the relationship between ERA and loneliness, structural equation modeling was used in AMOS. Two possible kinds of mediation are: full mediation and partial mediation. Full mediation is when there is a relationship between ERA and Loneliness such that, when the mediating variable is included, the ERA- Loneliness relationship completely disappears. Partial mediation is when there is a relationship between ERA and Loneliness such that, when the mediating variable is included, the ERA- Loneliness relationship weakens or changes in direction but still remains significant [67].

### Measurement Model

The measurement model included three latent variables, ERA, EA, and loneliness, and thirty-nine observed variables. All observed variables were loaded onto their respective latent factors, which followed the original design of the measures. A primary test of the measurement model indicated good fit to the data (χ^2^ (699, 500) = 1639.89, *p <* 0.001, χ^2^/*df =* 1.925, SRMR = 0.05, RMSEA = 0.04, CFI = 0.92). All factor loadings for the indicators onto their corresponding latent variables were significant (*p* < 0.05, indicating that all the latent factors were well represented by their respective indicators) (i.e. this supports that the measurement shows convergent validity, 63).

### Structural Model

**Fig 1:**
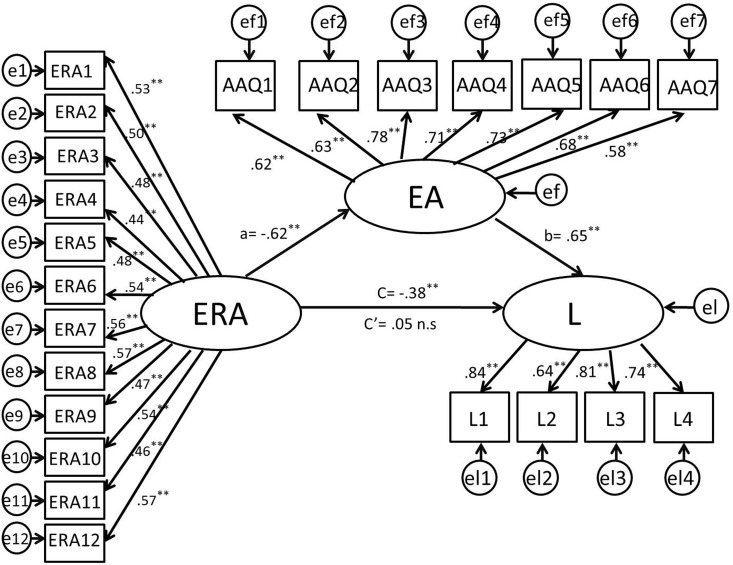
Final structural model (n = 500, standardized path coefficients). *Note*: *ERA* = emotion regulation abilities; *EA* = experiential avoidance; *L* = loneliness (*L1–L4* four parcels of loneliness). *p<0.05; **p<0.01.

We used maximum likelihood estimation in AMOS version 17.0 to test the structural model. First, we deleted all non-significant associations (*p* ≥ 0.05) and left only latent variables with significant associations in the model. The results shown in Fig 1 indicate that the direct standardized path coefficient from the predictor (failure-related action orientation) to the criterion (Loneliness) in the absence of mediators was significant, β = -0.40, *p* < 0.001. This mediated model exhibited a good fit to the data: χ^2^ (228, 500) = 437.519, *p <* 0.001, χ^2^/*df =* 1.6519, SRMR = 0.04, RMSEA = 0.04, CFI = 0.95. As the residuals from all the variables were not significant, no additional path was significant [62]. According to the mediation testing procedure (the path coefficients among these variables were all significant) [67] (Table 2), we found that EA was a full-mediator between ERA and loneliness. Therefore, these results assisted in our understanding of the role of EA in our hypothesized model. In addition, we tested an alternative model (EA—ERA—Loneliness; χ^2^ (227, 500) = 436.975, *p <* 0.001, χ^2^/*df =* 1.925, SRMR = 0.05, RMSEA = 0.05, CFI = 0.92), but we found that the path between ERA and loneliness was not significant (β = 0.05, *p* = 0.482).

**Table 2:** Direct and indirect effects and 95% confidence intervals for the mediation model.

Model pathways	Estimated effect	95% CI	
		Lower bounds	Upper bounds
*Direct effects*			
ERA—EA	-0.62^a^	-0.69	-0.55
EA—L	0.65^a^	0.58	0.70
*Indirect effects*			
ERA—EA—L	-0.40^a^	-0.46	-0.34

Note. n = 500; *ERA* = emotion regulation abilities; *EA* = experiential avoidance; *L* = loneliness

^a^ Empirical 95% confidence interval does not overlap with zero.

## Discussion

Although the connection between ERA and loneliness has been suggested by findings from previous research, the mechanisms through which these variables affect loneliness has not been well understood. This is the first study to explicitly show this relationship. Over decades ago, Hayes et al. (1996) proposed that many emotional distresses share a common experiential avoidance function. Although a growing literature has explored the effect of EA on psychological distress (see Hayes, Luoma, Bond, Masuda, & Lillis, 2006), few studies have investigated EA’s effect on loneliness. The present study was designed to understand this relationship more fully.

As expected, ERA was significantly and negatively related to feelings of loneliness and to EA. EA was also significantly and positively related to loneliness. These results supported our hypotheses and were consistent with previous findings related to emotion regulation and loneliness [15, 16, 49, 68]. Particularly, the current study suggested that, within Chinese culture, individuals with low levels of ERA were more likely to have a greater tendency to engage in EA. In addition, individuals low on ERA tended to have heightened feelings of loneliness.

Most of all, we tested a theoretically-grounded model wherein EA mediated the association between ERA and feelings of loneliness. As we expected, EA fully mediated the relationship between ERA and loneliness. The finding suggests that the extent to which ERA is related to loneliness may be explained by individuals’ different tendencies to willingly remain in touch with unwanted private experiences. According to Action Control Theory, ERA is conceptualized as a trait-like characteristic representing the ability to regulate emotions, cognitions, and behaviors involved throughout the process of accomplishing a goal. Especially in threatening situations, high ERA individuals could be more effective in down-regulating stress and negative emotions compared to low ERA individuals. Thus, as a consequence, low ERA persons may tend to ruminate about negative experiences long after they occur [69]. In that case, ERA may, therefore, help individuals feel less lonely through their use of emotion regulation strategies. Specifically, low ERA individuals may have a greater tendency to engage in EA. Although adopting an EA strategy may help individuals succeed in down-regulating initial discomfort, people would probably ultimately experience a rebound effect with increased distress [70]. Since controlling thoughts and suppressing unwanted emotions can be cognitively draining, high EA may potentially leave people impaired during social interaction [42]. This is because people with high EA will pay less attention to others [43] and they may be perceived as less engaged [44]. The result is that high EA people may suffer from higher levels of loneliness. This is consistent with evolutionary perspectives of loneliness.

Overall, these findings highlight mechanisms underlying the relationship between ERA and loneliness, and they suggest that EA may be an important target for intervention strategies for lonely individuals. Moreover, some limitations of this study should be acknowledged. First, this study adopted a cross-sectional, correlational design, which allows only for cautious interpretation of results, as alternative models could also be possible. The cross-sectional design of this study precludes inferences regarding the direction of associations between variables. Thus, longitudinal or experimental studies could facilitate causal evaluations of similar phenomena in the future. The second limitation of our study is that our sample is unlikely to be representative of the Chinese young adult population: the study was only conducted in one school in China. Its results may not be generalizable to other populations. Furthermore, item parcels were formed to create latent values loneliness constructs. The use of item parceling in SEM offers advantages, but parceling has potential drawbacks such as it may increase the chance of Type II error [66]. Lastly, this study exclusively used self-report questionnaires for data collection. Future studies could use multiple data sources for evaluation (e.g., observer reports, behavioral measures, or data from experimental manipulations).

## Conclusion

Despite limitations, our findings contribute to the existing literature on the factors associated with ERA, and they provide an empirical framework for researchers to test the mediating effects of EA on the relationship between ERA and loneliness. The findings suggest EA is a key mechanism in explaining why people with high ERA are prone to feeling lower levels of loneliness. In particular, this study’s findings have important implications for designing effective psychological interventions for loneliness, such as interventions that target EA (e.g., Acceptance and Commitment Therapy, ACT). Future research might investigate the potential effectiveness of ACT as an intervention for chronic loneliness.

## Supporting Information

S1 Dataset Raw data of experiential avoidance, emotion regulation abilities and loneliness. (SAV)
